# A novel, PCP‐dependent tissue organising principle coordinating morphogenesis between embryonic skin epidermal layers

**DOI:** 10.1111/joa.70099

**Published:** 2026-01-08

**Authors:** C. J. Formstone

**Affiliations:** ^1^ Centre for Development Neurobiology King's College London London UK; ^2^ Present address: Department of Clinical, Pharmaceutical and Biological Sciences, School of Health, Medical and Life Sciences University of Hertfordshire Hatfield UK

**Keywords:** cell long axis orientation, epidermis, PCP, rosette, secondary body wall, tissue polarity

## Abstract

Planar cell polarity (PCP) provides cells and tissues with a sense of direction in relation to the principal body axes of the embryo. In the developing mouse skin, PCP coordinates cell behaviours within the plane of the epidermal basal monolayer. In this report, evidence is presented for a novel, three‐dimensional PCP protein‐dependent tissue organising principle(s) operating within the mouse embryonic epidermis which coordinates cell long axis orientation across multiple epidermal layers. Here, the core‐PCP protein, Frizzled‐6 (Fz6), is found within different layers of developing trunk epidermis. Analysis of *fz6* mouse mutant skin suggests Fz6 signalling contributes to several aspects of the novel tissue organising principle. Firstly, the robust coordination of epidermal cell long axis orientation between epidermal layers. Secondly, the timing of the switch in epidermal cell long axis orientation between orthogonal principal body axes, circumferential and longitudinal. Finally, the establishment of robust mirror symmetry of epidermal cell long axis orientation between each mouse embryo mid‐flank, when viewed across the ventral midline. Local cell arrays/cell rosette‐type arrangements within adjacent epidermal layers are implicated in the underlying mechanism coordinating epidermal cell long axis orientation. A previously unreported morphogenetic event within the superficial layers of the nascent epidermis may also rely on three‐dimensional tissue polarity processes.

## Graphical abstract

‘Outside‐in’ expression of Fz6 across multiple layers of mouse embryo epidermis is connected to Fz6 asymmetry in the basal layer plane. Fz6 loss‐of‐function correlated with failure to: coordinate axial bias of cell long axis orientation across epidermal layers, switch axial cell long axis orientation during suprabasal layer development and establish suprabasal layer 5‐cell rosettes, known to change cell orientation in the tissue plane.

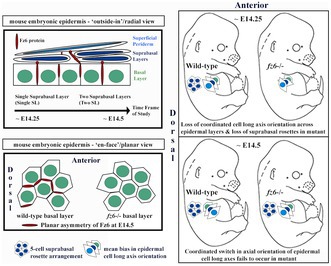

## 1 INTRODUCTION

Interaction between different tissue layers is essential for efficient organ formation in vertebrates (Biggs & Mikkola, 2014; Branco et al., 2022; Morita et al., 2017). French et al. (1976) however posited that pattern formation in animal organs might generally be determined in two dimensions rather than in three dimensions because of the availability of inductive signalling (Arkell & Tam, 2012; Mangold & Spemann, 1927) and epithelial to mesenchymal transition (Moore Zajic et al., 2025; Thiery, 2003).

Here, evidence is presented for a novel three‐dimensional tissue organising principle acting across the mouse skin epidermis which coordinates directional tissue morphogenesis between epidermal layers (inter‐tissue interaction) as the epidermis encloses the embryonic trunk. Mouse trunk epidermis is established at each mid‐flank around E13.5 (Panousopoulou et al., 2016) and subsequently spreads dorsal ward and ventral ward as part of the enclosure process of the developing secondary body wall (SBW; reviewed in Formstone et al., 2024). ‘Outside‐in’ (radial) intercalation of a thickened basal epidermal layer generates an organised basal monolayer (BM) beneath a superficial epithelial layer, called the periderm. Radial intercalation of basal cells concomitantly spreads the epidermis outwards from the mid‐flank (Panousopoulou et al., 2016). Soon after, suprabasal cells emerge between the basal and periderm layers via delamination of basal progenitors (Damen et al., 2021; Miroshnikova et al., 2018; Williams et al., 2014). Once established, the suprabasal layer progressively thickens and finally begins to differentiate (Hanson, 1947).

Notably, the epidermis is built as the SBW encloses the embryo trunk, raising questions of how coordination of morphogenesis between epidermal layers and between the epidermis and the SBW is achieved. Here it is reported that cell long axis orientation is coordinated between epidermal layers in wild‐type skin and sequentially aligns along two orthogonal body axes, first circumferential (anterior‐ventral) and then longitudinal (anterior‐dorsal), from E14.25 to E14.5. Mirror symmetry of epidermal cell long axis orientation is concomitantly generated between each mid‐flank of the developing SBW. Each of these characteristics is disrupted in *fz6* mouse mutant skin. Data suggest rosette‐type cell–cell arrangements as a plausible underlying cellular mechanism for the Fz6‐dependent, three‐dimensional tissue organising principle described.

## 2 RESULTS

### 2.1 Cell long axis orientation is robustly coordinated across multiple epidermal layers during generation of the suprabasal layer in wild‐type mouse embryos

**FIGURE 1:**
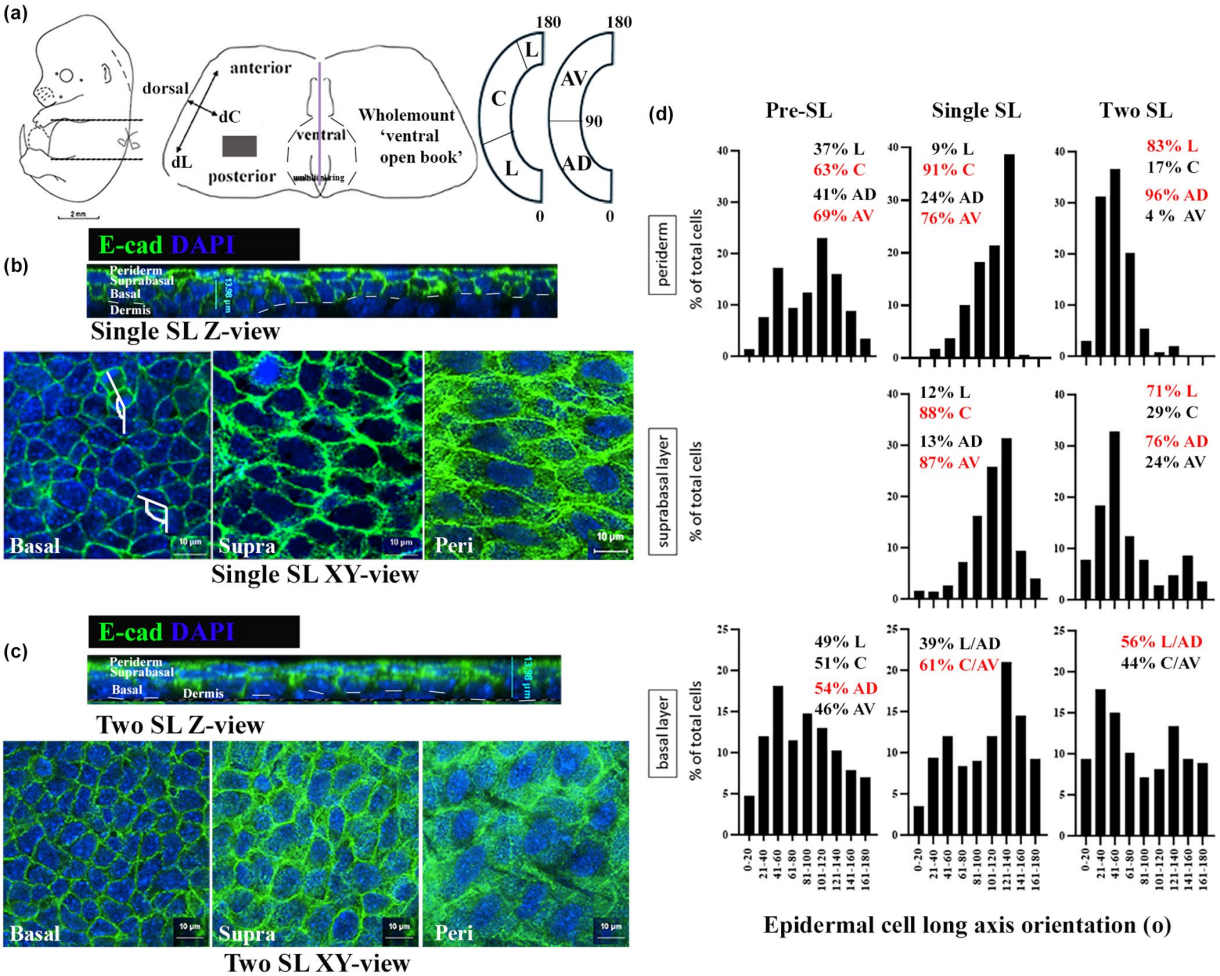
A coordinated switch in the mirror symmetric orientation of epidermal cell long axes from circumferential to longitudinal occurs across epidermal layers as the suprabasal layer thickens from one to two layers. (a) Left, lateral view schematic, taken from the Kaufman Atlas (Edited by Richard Baldock, Duncan Davidson, Jonathon Bard and Gillian Morris‐Kay, published by Elsevier) of a mouse embryo staged E14–E14.5. Dotted horizontal lines label anterior (top) and posterior (bottom) margins of dissected body wall. Scissors mark line of dissection along edge of spinal cord on the dorsal side. Centre, schematic showing ventral open book preparation of wholemount immunostained mouse embryo body wall. Grey box shows a representative mid‐flank location on embryo's right‐hand side (left‐hand side when viewed *en‐face*) from where confocal images were taken for data collection. Measurements of cell long axis and cell division orientation were taken relative to the ventral midline, indicated by a vertical mauve line. Position of the sternum (anterior) and umbilical ring (posterior) are shown at the ventral midline. Dashed back lines indicate presumptive position of the leading edge of the epidermis on the ventral side around E14.25–E14.5, unpublished data also suggests that dorsal epidermis is progressively covering the spinal cord at this time. The definitive longitudinal axis (dL) was defined by the orientation of the dorsal edge of the open book wholemount when compared to the ventral midline. Circumferential (dC) is perpendicular to the longitudinal axis. Both axes are indicated by double‐headed arrows. Right, schematics of quadrants used to decipher axial bias. (L) defines longitudinal orientations (161° through to 70°), (C) defines circumferential orientations (71 to 160°). AV defines anterior‐ventral orientations (90–180°) and AD defines anterior‐dorsal orientations (0–90°). (b, c) Z‐stack and XY‐views of immunostained wild‐type epidermis with a single suprabasal layer (b, Single SL) or with two suprabasal layers (c, Two SL), scale bars are shown. XY views, anterior is to the top and dorsal to the left in all images. White dashed lines in Z‐stack views (b, c) label the basal lamina between the epidermis and dermis. Strategy for measurement of cell aspect ratio (lower angle schematic) and planar orientation of cell division (upper angle schematic) are shown on image of the basal layer (Basal) in (b). Supra, suprabasal; peri, periderm. (d) Histograms show the percentage of total epidermal cells allocated to specific bins of 20° width which specify the orientation of longest cell axis measured relative to the ventral midline (see schematic in (a)). Percentage (%) of longitudinal (L), circumferential (C), AV and AD orientations are shown, value in red highlights the strongest axial orientation bias. Embryos were grouped according to suprabasal layer development. The first group includes embryos who have not established a single suprabasal layer (Pre‐SL), staged around E14, outer‐most superficial layer was imaged and scored. The second group had established a single suprabasal layer (Single SL) and were staged around E14.25. The final group had thickened their suprabasal layer (Two SL) and were staged around E14.5. Total numbers of cells scored at each stage are provided in the methods section. (b–d) Wild‐type, C57BL6 background, *n* = 5 biological replicates from *n* = 2 litters at E14, *n* = 5 biological replicates from *n* = 4 litters at E14.25 and *n* = 4 biological replicates from *n* = 3 litters at E14.5.

To address the question of whether directional morphogenesis across basal, suprabasal and periderm epidermal layers is coordinated during SBW enclosure, epidermal cell long axis orientation (LAO), cell aspect ratio, as well as the orientation of epidermal cell division were investigated in the same set of wild‐type immunostained, ventral open book SBW wholemounts (Figure 1a). Epidermis was analysed during the generation of the suprabasal layer. Individual embryos who were poised to, or had started to, generate a suprabasal layer were staged by plug date as approximately E14 (Pre‐SL). Individual embryos who had generated a single suprabasal layer were similarly staged as approximately E14.25 (see representative Z‐stack image, Figure 1b, single SL). Individual embryos who had generated two suprabasal layers were staged as approximately E14.5 (see representative Z‐stack image, Figure 1c, two SL). Measurements were made from both the right‐hand (RHS) and left‐hand (LHS) mid‐flank of each mouse embryo (grey box, Figure 1a, shows an example of the area of analysis within the RHS flank of a mouse embryo, which is positioned on the left when wholemounted ventral peels are viewed *en face*). For consistency, where suprabasal layer (SL) measurements were made, only suprabasal cells directly overlying the BM were scored.

Strategies for measurement of cell long axis and planar cell division orientations are shown in Figure 1b (basal). Dorsal for each flank was always positioned to the left and cell orientations were measured clockwise from 0°, which defines the ventral midline (marked by a vertical mauve line, Figure 1a). Cell orientation measurements (0 to 180°) were placed into bins, each spanning 20°. The percentage of the total number of cells in each bin was plotted as a histogram (Figure 1d). Longitudinally (L) and circumferentially (C) orientations were defined because the E14–E14.5 embryo trunk is effectively a cylinder (Figure 1a). To generate ventral open book wholemounts, each flank was dissected along the length of the developing spinal cord (Figure 1a). Thus, the dorsal edges of each wholemount were viewed as representing an anterior–posterior axis of the developing embryo and thus defined the definitive longitudinal axis (dL; Figure 1a). This axis, when viewed *en‐face* in ventral open book wholemounts, was estimated to be around 25° away from the ventral midline. Orientations ±45° to dL were thus deemed as longitudinal (L), that is, 161° through to 70°. The definitive circumference (dC) of the cylindrical embryo trunk is perpendicular (90°) to longitudinal, which is 115° to the ventral midline (Figure 1a). Orientations ±45° to dC were therefore defined as circumferential (C), that is, 71 to 160°. From an anatomical viewpoint, however, cell orientations of 0–90° and 91–180° to the ventral midline can be considered as anterior‐dorsal (AD) or an anterior‐ventral (AV), respectively (Figure 1a). On each histogram, therefore, the percentage of longitudinal (L) and circumferential (C) as well as AD and AV orientations are shown: percentages for AD/AV are shown only if different to %L and %C (Figure 1d). The strongest axial bias is highlighted in red (Figure 1d).

Prior to/during initial stages of suprabasal layer establishment, the mean percentage of basal cells in circumferential versus longitudinal bins did not reveal any specific bias in LAO, whereas AD orientations were slightly more prevalent than AV (Pre‐SL; Figure 1d, *n* = 5 embryos). Conversely, overlying superficial periderm cells were inclined towards circumferential/AV axes (Figure 1d). Subsequent analysis of individual embryo flanks (*n* = 10) revealed that an equal number exhibited either a circumferential/AV bias or a longitudinal/AD bias for basal cell LAO, while for periderm cells, 5/10 mid‐flanks exhibited a circumferential/AV bias and 3/10 a longitudinal/AD bias (Figure S1A). Robust coordination of LAO between basal and periderm layers was observed in 8/10 mid‐flanks (Figure S1A). Notably, both clock‐wise (*n* = 2 embryos) and anti‐clockwise (*n* = 1) chirality of periderm LAO at opposing flanks was apparent, as well as two instances of mirror symmetry (Figure S1B).

Upon establishment of a single suprabasal layer, LAO was consistently biased towards circumferential/AV across all epidermal layers, being particularly robust in suprabasal and periderm layers (single SL: Figure 1d; Figure S2A). Figure 1b shows representative images of wild‐type epidermis exhibiting a single suprabasal layer. Mirror symmetry of LAO alignment between opposing flanks was also consistently observed (Figure S2B). In one embryo (#3), basal cells were biased towards longitudinal/AD (Figure S2A,B) whereas suprabasal and periderm cell LAO were predominantly within circumferential/AV bins (Figure S2A,B). A similar longitudinal/AD shift was also perceptible in embryo #2 (RHS). These differences were of interest because once the suprabasal layer had thickened to two layers, epidermal LAO switched bias, in a coordinated manner, towards the longitudinal axis (Two SL; Figure 1d; Figure S2C,D). Figure 1c shows representative images of wild‐type epidermis exhibiting two suprabasal layers. Mirror symmetry of LAO was retained despite the switch from circumferential/AV towards longitudinal/AD orientations (Figure S2D).

Altogether these data reveal the emergence of a robust coordination of epidermal LAO directed along the circumferential/AV axis of the developing trunk once the suprabasal layer is established. Subsequently LAO switches to a longitudinal/AD bias as the suprabasal layer thickens: coordination of LAO was observed across all epidermal layers at each mid‐flank.

**FIGURE 2:**
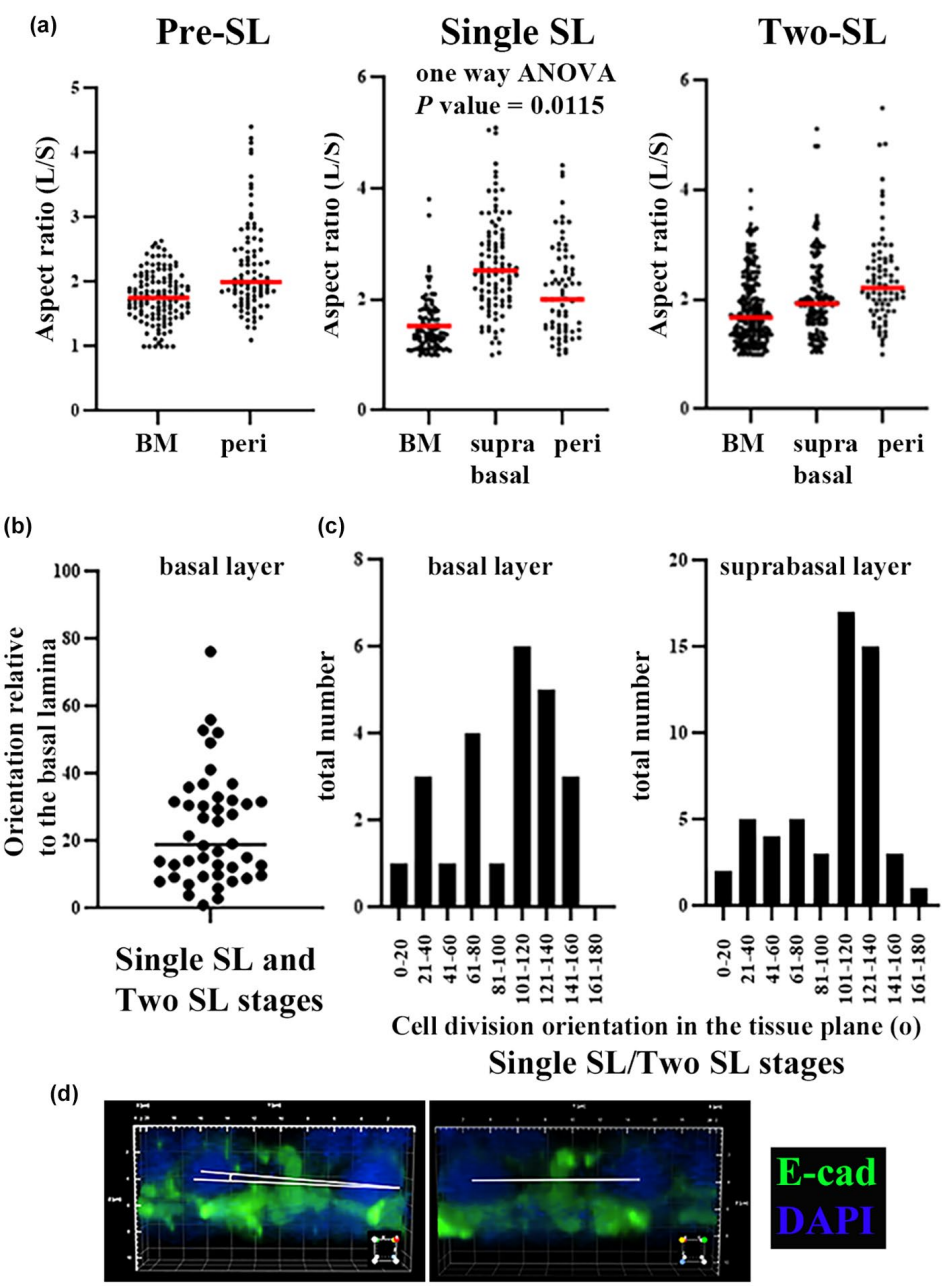
Planar orientation of cell division in wild‐type remains biased towards circumferential as the suprabasal layer thickens from one to two layers. (a) BM denotes basal monolayer, peri denotes periderm. Each dot represents the aspect ratio value (length of longest cell axis divided by length of short cell axis) for an individual epidermal cell at embryonic epidermal stages shown, Pre‐SL is around E14, Single SL is around E14.25 and Two SL is around E14.5. Horizontal red solid line shows the mean value for each epidermal layer. Biological replicates were subjected to statistical analysis using one‐way ANOVA with Tukey ad‐hoc test. *p* Values are shown. Multiple comparison *p*‐values: single SL basal/suprabasal = 0.011, two SL basal/suprabasal = 0.024, basal/peri = 0.007 (b) Orientation of telophase cell divisions in relation to the basal lamina (^o^) is shown for *n* = 44 basal monolayer cells in telophase scored from *n* = 20 mid‐flank images from wild‐type BALB/c mouse embryos (Single SL and Two SL data were combined). (c) Total number of basal and suprabasal telophase divisions with planar orientations at binned angles relative to the ventral midline scored from the same mid‐flank images as used for (b). Basal telophase divisions from total basal cells scored in (b) were analysed if they exhibited an orientation relative to the basal lamina of 20° or less (*n* = 24). Suprabasal telophase divisions were scored from the same twenty wild‐type mid‐flank images described in (b): *n* = 55. (d) 3D cropped images of the same telophase planar cell division, views from each lateral face of the cell division are shown, as is the strategy for measuring angle of chromatid separation relative to the basal/dermal interface, which is labelled by E‐cadherin (green).

Measurement of cell aspect ratio revealed hyper‐extended suprabasal cell long axes, compared to basal cells, across wild‐type epidermis exhibiting a single suprabasal layer compared to epidermis with a thickened suprabasal layer (Figure 2a; see Figure 1b (basal) XY view for measurement strategy). Measurement of planar cell division orientation (telophase divisions) revealed circumferential/AV bias across basal and suprabasal layers at each stage analysed (Figure 2c). Periderm divisions were not scored due to their low numbers, but when observed, they also aligned circumferentially/AV. Indeed, all planar divisions across epidermis with a single suprabasal layer aligned with the circumferential/AV axis (100%, *n* = 24). Longitudinal/AD orientations emerged however once the suprabasal layer had thickened (29%, *n* = 9/31). Measurement of basal cell division orientation along the Z‐axis (analysed from 3D images of each cell division using cropped confocal Z‐stacks of wholemounted skins, Figure 2d) revealed a mean orientation relative to the basal lamina of 20° (Figure 2b).

Altogether, these data reveal that LAO and planar‐oriented cell division (POCD) are not always coupled as the mouse epidermal suprabasal layer is generated. Increased suprabasal cell aspect ratio within the single suprabasal layer may promote circumferentially oriented growth of the suprabasal layer as it is established.

### 2.2 Periderm cell interfaces manifest planar polarity prior to emergence of robust Fz6 asymmetry in the epidermal basal layer

Molecular pathways of planar polarity orient the directional alignment of cell behaviours across epithelial tissues (reviewed by Wallingford, 2012) and include E‐cadherin/Par3/Myosin II (Bertet et al., 2004; Blankenship et al., 2006; Zallen & Wieschaus, 2004), Fat/Dachsous proteins (Mao et al., 2013; Trinidad et al., 2025) and Frizzled/Celsr1/Vangl core‐PCP pathway (Cetera et al., 2018; Nishimura et al., 2012; Rawls & Wolff, 2003; Ybot‐Gonzalez et al., 2007). A unifying feature of each pathway is the asymmetric enrichment of protein components to opposing cell interfaces along specific body axes (Axelrod, 2001; Brittle et al., 2012; Devenport & Fuchs, 2008; Lawrence & Casal, 2018; Oozeer et al., 2017; Usui et al., 1999). The core‐PCP pathway defines both long‐range and short‐range planar polarity across the mouse embryonic epidermis (Chang et al., 2016; Devenport & Fuchs, 2008; Oozeer et al., 2017). Importantly, the presence of robust, long‐range, anterior–posterior (longitudinal) oriented Fz6 asymmetry across the epidermal basal layer is reported to be established by E14.5 (Devenport & Fuchs, 2008), raising the question of whether core‐PCP signalling plays a role in the switch in epidermal cell long axis orientation from circumferential to longitudinal at the same embryonic stage.

**FIGURE 3:**
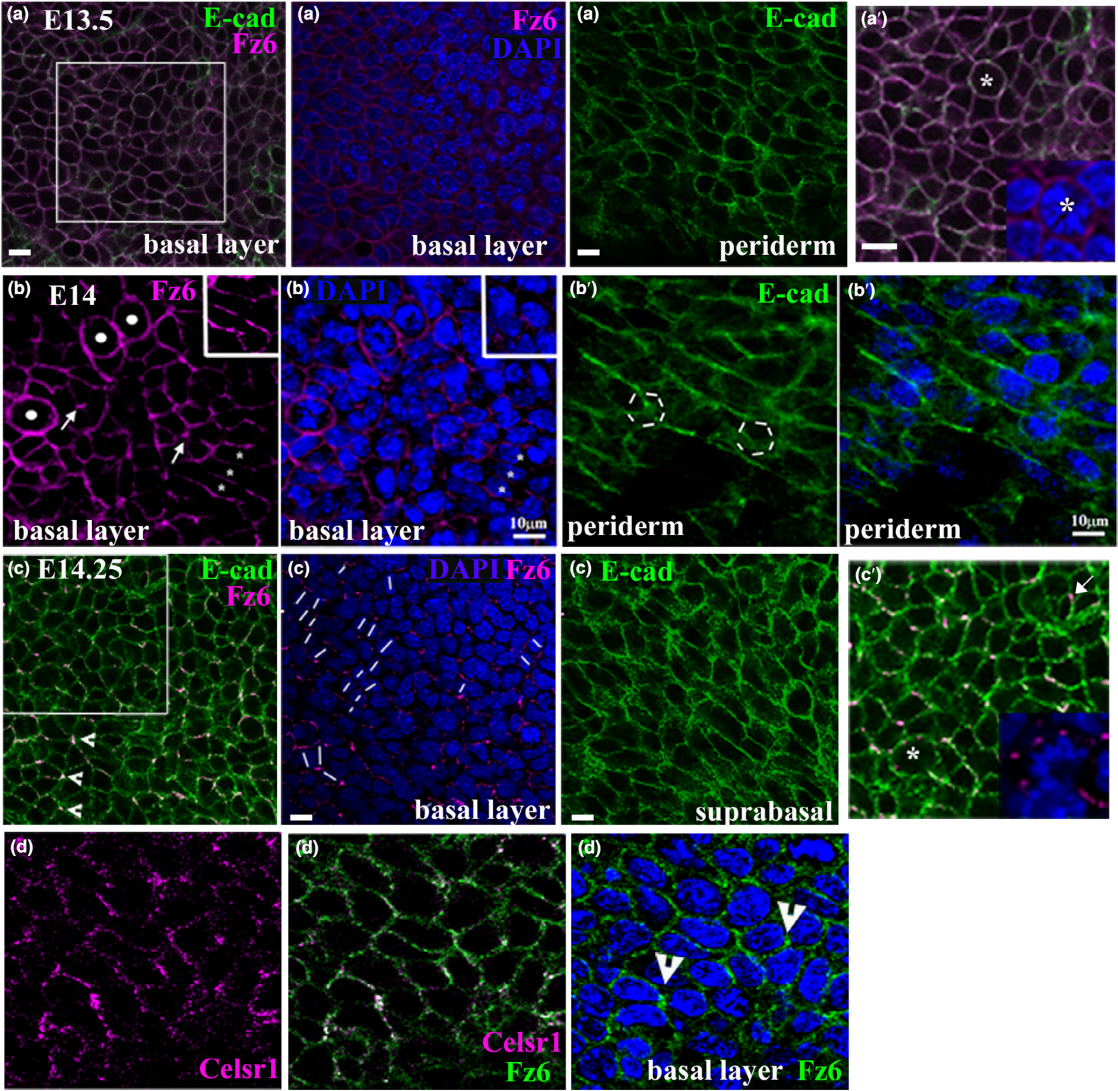
Evidence for A‐P oriented planar polarisation of E‐cadherin within the periderm prior to emergence of robust basal layer Fz6 planar polarity. Scale bar is 10 μm throughout. Ventral is towards the right and posterior towards the bottom in all images which are representative of at least 3 embryos from *n* = 2 wild‐type litters. (a–c) Wholemount co‐immunostaining of Fz6 and E‐cadherin in mid‐flank wild‐type epidermis. (a, a′) XY view. Area within white box is shown magnified in (a′). Asterisk in (a′) labels dividing basal cell during prophase, combined DAPI and Fz6 labelling of dividing cell is shown magnified further in bottom right‐hand corner of (a′). (b, b′) XY view. Fz6 is observed at the centre of rosette‐type basal cell arrangements, planar polarised E‐cadherin (Ecad) expression is observed across E14 periderm (*n* = 6/10 embryos assessed for E‐cadherin expression), outer most layer of superficial periderm cells was imaged. Arrowheads label Fz6 puncta associated within other less well‐defined basal cell arrangements. Small white asterisks label a single instance where robust Fz6 planar polarised asymmetry within the basal monolayer was found alongside planar polarised E‐cadherin expression, insets top right show magnified views. White dots label basal cells in prophase. (b′) Circular dashed lines label position of underlying Fz6‐positive basal layer rosettes. (c, c′) XY view. Tissue within white box is shown magnified in (c′). (c) White lines label orientation of local areas of planar polarised Fz6 expression across the basal monolayer. Arrowheads label Fz6 expression in the centre of rosette‐type cell arrangements. (c′) White arrow (top right) labels Fz6 enrichment at the centre of a 4‐cell array, asterisk labels internalisation of Fz6 during prophase. Inset bottom right shows magnified view of basal cell in prophase highlighted by asterisk, DAPI and Fz6 staining (d) Wholemount co‐immunostaining of Fz6 and Celsr1. XY view. Arrowheads label expression of Fz6 in the centre of rosette‐type cell arrangements which co‐localise with Celsr1 expression. Notably, the left‐hand arrowhead highlights Celsr1 enrichment along a single central interface between opposing neighbours, appearing as if in a 4‐cell array.

As a first step to address this question, the expression pattern of the core‐PCP protein Frizzled‐6 (Fz6) was investigated by wholemount immunostaining. *En‐face* views of E13.5 skin, when the epidermis consists of a BM overlain with superficial cells (periderm), revealed symmetric Fz6 expression at basal cell interfaces during interphase (Figure 3a‐a′) and prophase (insert, Figure 3a′). Localised Fz6 enrichment at cell interfaces was evident by E14 (Figure 3b), just prior to emergence of suprabasal cells, although robust planar polarised Fz6 asymmetry was rarely observed (small white asterisks in basal layer, Figure 3b). Fz6 was additionally found enriched at the centre of basal cell rosette‐type arrangements (arrows, Figure 3b), including 4‐cell arrays (arrow, Figure 3c′).

Intriguingly, Fz6‐positive basal cell rosettes/4‐cell arrays were sometimes overlain by areas of planar polarised E‐cadherin staining, where E‐cadherin enriched to anterior–posterior (A‐P)‐oriented cell interfaces of rectangular‐shaped superficial (periderm) cells (Figure 3b,b′; Figure S3A). Overlap between basal cell Fz6 planar polarised asymmetry and superficial planar polarised E‐cadherin expression was however rarely observed (asterisks, Figure 3b, *n* = 1 of 10 E14 wild‐type skins imaged). In the Drosophila germ band, epithelial asymmetry of E‐cadherin controls asymmetry of Myosin II flows and determines the strength of coupling at cell–cell junctions undergoing cell intercalation (Levayer & Lecuit, 2013). Vinculin is also a component of E‐cadherin‐containing adherens junctions (AJs) which regulates the strength of AJ coupling (Morales‐Camilo et al., 2024). Wholemount co‐immunohistochemistry of E14 mouse embryonic epidermis revealed vinculin enriched to anterior–posterior (A‐P) oriented superficial cell interfaces (Figure S3B) associated with puncta containing phosphorylated Myosin II regulatory light chain (pMRLC) (short white arrows, lower right‐hand image Figure S3B), a marker of active acto‐myosin (Watanabe et al., 2007). pMRLC puncta were also observed in the centre of underlying basal layer rosette‐shaped cell arrangements (long white arrow, lower left‐hand image Figure S3B).

When a single epidermal suprabasal layer had been established, Fz6 expression was found strongly punctate at basal cell interfaces (Figure 3c). Local areas of A‐P‐oriented planar polarised Fz6 asymmetry were more frequently observed (white lines denote axis of Fz6 asymmetry, Figure 3c) and Fz6 protein was now internalised during prophase (inset, bottom right, Figure 3c′) as previously reported (Devenport et al., 2011). Fz6 expression at the centre of rosette‐type basal cell arrangements was maintained through epidermal development (Figure 3d), co‐immunohistochemistry revealed co‐localisation of Fz6 with the core‐PCP protein, Celsr1 (arrowheads, Figure 3d).

Altogether the data above show emergence of robust A‐P oriented Fz6 planar polarised asymmetry across the epidermal BM following establishment of a single suprabasal layer. Prior to this stage, Fz6‐expressing basal cell arrays/rosette‐type arrangements locate directly beneath a novel planar polarised expression of E‐cadherin, vinculin and pMRLC within the most superficial epidermal layer.

### 2.3 Morphogenesis of the superficial layers of the E14 epidermis is dependent on Fz6 signalling

**FIGURE 4:**
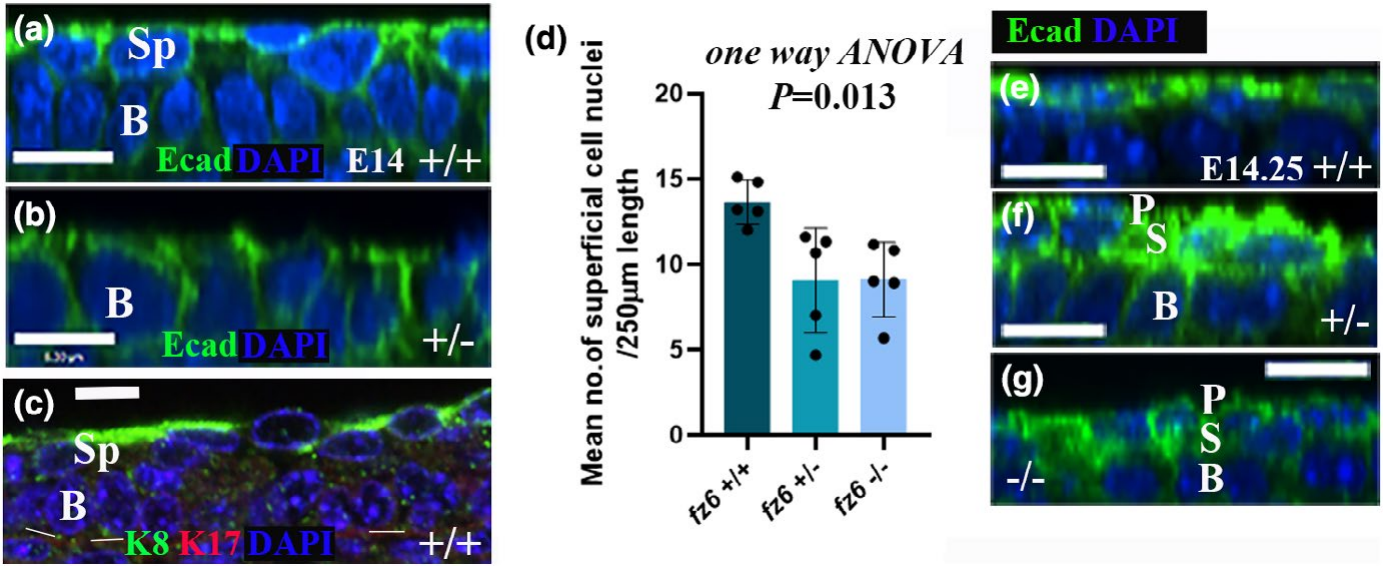
The architecture of superficial epidermal layer(s) is disturbed in E14 *fz6* mutant epidermis. (a, b, e–g) Representative confocal Z‐stack images, scale bars are 10 μm throughout. Sp denotes superficial layers, P denotes periderm, B denotes basal monolayer, S denotes single suprabasal layer. +/+ denotes wild‐type, +/− denotes heterozygote *fz6* knockout, −/− denotes homozygote *fz6* knockout from the same litter, *n* = 5 embryos from 4 litters for each condition. (c) Wax section of wild‐type (+/+) E14 epidermis stained with K8 (green) and K17 (red). No signal was observed with K17 antibody in wax sections (with or without antigen retrieval) or frozen sections from E14 wild‐type embryos (data not shown). Keratin1/Keratin10 staining was also not observed in frozen sections (data not shown). K8 staining in superficial cells was observed in frozen sections (data not shown). Scale bar is 10 μm, white dashed lines label the basal lamina (d) Mean number of superficial cells (from 6 technical replicates per embryo) were measured along a specified length of epidermis from Z‐views of confocal images. Biological replicates (*n* = 5) were submitted to one way ANOVA with a Tukeys ad hoc test, *p* value is shown.

Enrichment of Fz6 within basal rosettes underlying A‐P‐oriented peridermal E‐cadherin cell surface enrichment raised the question of whether Fz6 signalling played a role in the planar polarised distribution of peridermal E‐cadherin. To address this question, E14 mid‐flank skins from *fz6* mutants (*fz6+/−* and *fz6−/−*; Guo et al., 2004) were analysed. Unexpectedly, however, both *fz6+/−* and *fz6*−/− mutants exhibited disturbed architecture of the superficial epidermal layers; indeed, the epidermal basal monolayer was often found ‘naked’ with few overlying superficial cells (representative images, Figure 4a,b: *n* = 5 embryos from 4 litters for each condition). Quantification of superficial cell numbers demonstrated a significant reduction in superficial cell density in mutant embryos compared to wild‐type (Figure 4d). The identity of the superficial cells lost in *fz6* mutants is, however, unclear. Keratin 1 and Keratin 10, which mark suprabasal cells (Damen et al., 2021), labelled superficial cells at E14 in wax sections (with antigen retrieval) from wild‐type mouse epidermis (Panousopoulou et al., 2016) but produced no signal in frozen sections taken at the same embryonic stage (data not shown). Keratin‐17 (K17), which labels periderm at around E14–E14.5 (Hammond et al., 2019; McGowan & Coulombe, 1998), did not produce any specific signal in either E14 wax or frozen sections (Figure 4c). Keratin‐8 (K8), however, a marker for surface ectoderm (Shalom‐Feuerstein et al., 2011), was found to label the most superficial E14 epidermal cells (Figure 4c) in both wax and frozen sections, consistent with Jacob et al. (2023). Notably, Panteleyev (2022) raises the hypothesis that the mouse embryonic periderm is actually a bilayer, with an outer periderm layer comprising flat cells underlain by an inner peridermal layer with large nuclei. This description is strikingly similar to the organisation of the superficial cells shown in Figure 4a. Thus, a more detailed study of periderm morphogenesis is needed in the future to resolve the identity of the superficial cells affected by disrupted *fz6* function. Nevertheless, the data presented here raise the hypothesis that the superficial cell layers of the nascent epidermis undergo a hitherto unknown phase of morphogenesis prior to formation of the suprabasal layer, which is sensitive to wild‐type levels of Fz6 signalling. The unusual temporary loss of superficial epidermal cells in *fz6* mutants suggests failure of a primary morphogenetic event, which is then recovered as epidermal development proceeds.

### 2.4 Fz6 plays a role in the coordination of cell long axis orientation across the epidermis as the suprabasal layer is established

The emergence of robust A‐P‐oriented Fz6 asymmetry in the epidermal basal monolayer during generation of the suprabasal layer supported the hypothesis that Fz6 may play a role in the switch in epidermal cell long axis orientation bias from circumferential to longitudinal. As a majority of *fz6*+/− and *fz6*−/− embryos recovered periderm formation and went on to establish a single suprabasal layer around the same time as wild‐type littermates (Figure 4e–g), epidermal cell long axis orientation (LAO) was subsequently investigated in *fz6* knockout littermates. The same circumferential/AV and longitudinal/AD quadrants were used as for wild‐type.

**FIGURE 5:**
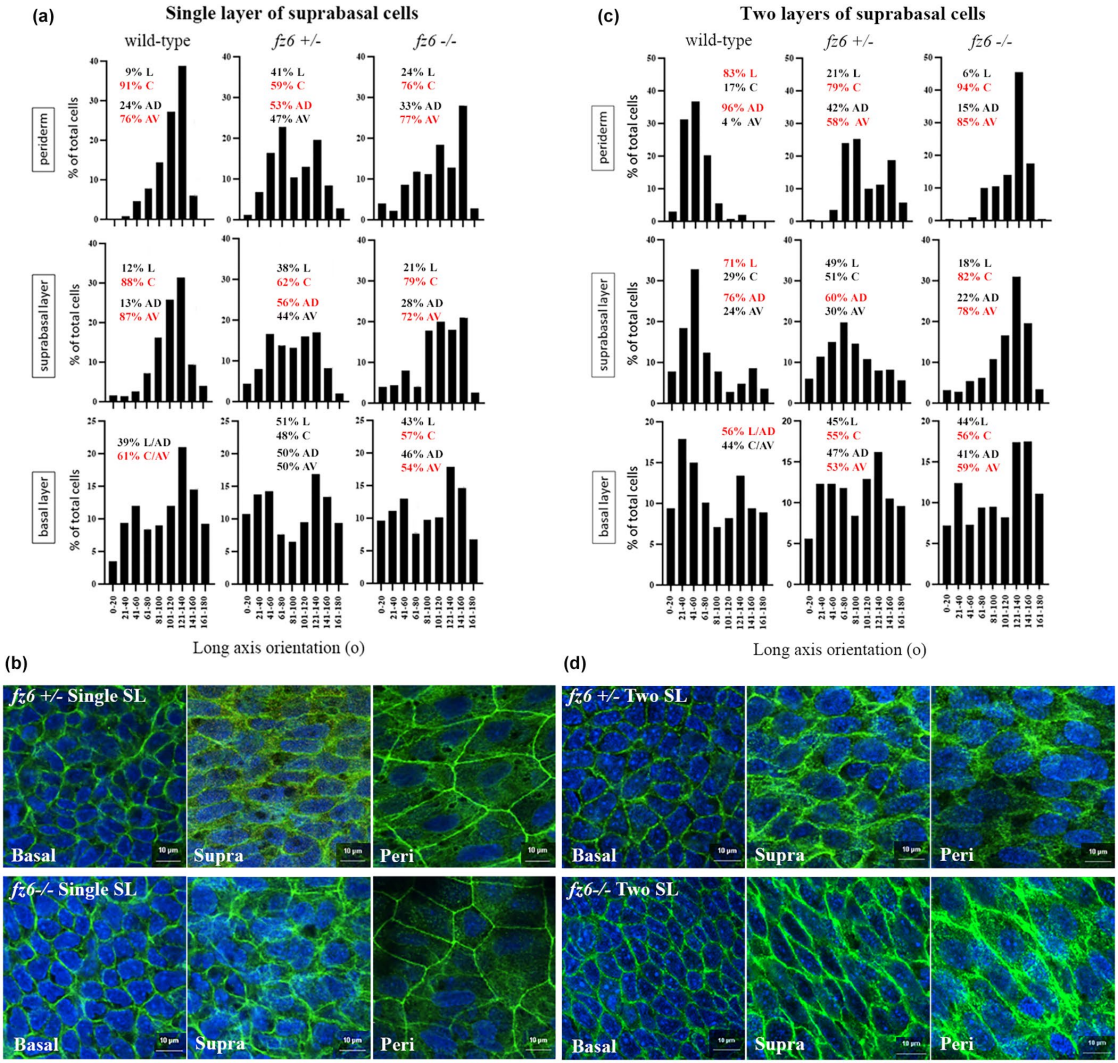
Robust coordination of epidermal cell long axis orientation and timing of changes in axial bias is disrupted in *fz6* mouse mutants. C57BL6 background, wild‐type data is the same as presented in Figure 1. Histograms show the percentage of total epidermal cells allocated to specific bins of 20° width which specify the orientation of the longest cell axis measured relative to the ventral midline (see schematic in Figure 1a). % of longitudinal (L) and circumferential (C) and anterior‐ventral (AV) and anterior‐dorsal (AD) orientations are shown, value highlighted in red represents the strongest orientation bias. Embryos were grouped according to suprabasal layer development as outlined in Figure 1. (a) Littermates had established a single suprabasal layer and were staged as around E14.25. Wild‐type, *fz6+/−* and *fz6−/−* embryos, *n* = 5 biological replicates each condition from 3 litters, total cells scored are detailed in methods. (c) Littermates had thickened their suprabasal layer (two layers) and were staged as around E14.5. Wild‐type, *fz6+/−* and *fz6−/−* embryos, *n* = 4 biological replicates from at least 3 litters, total cells scored are detailed in methods. (b, d) XY‐views of immunostained mutant epidermis with a single suprabasal layer (b, Single SL) or with two suprabasal layers (d, Two SL). Scale bars are shown, anterior is to the top and dorsal is to the left in all images. Supra, suprabasal; peri, periderm. Cell outlines are denoted by E‐cadherin immunostaining and cell nuclei by DAPI.

In embryos which had established a single suprabasal layer, overall, basal LAO was unbiased in *fz6+/−* skins and was less robust in *fz6−/−* skins compared to wild‐type (*n* = 5 embryos, each condition; Figure 5a; representative images are shown in Figure 5b). Analysis of individual embryos, however, revealed that basal cells from 3 out of 5 *fz6+/−* embryos exhibited bias towards longitudinal/AD axes at both mid‐flanks (Figure S4A). Conversely, basal cells from 6 out of 8 individual *fz6−/−* mid‐flanks exhibited circumferential/AV bias (Figure S4C). Suprabasal and periderm layers from *fz6*+/− embryos exhibited a broader spread of orientations than both wild‐type and *fz6−/−* skins (Figure 5a). Indeed, the coincident bias towards both circumferential and AV quadrants observed in wild‐type across epidermal layers was disrupted in *fz6*+/− skins (Figure 5a). Analysis of individual *fz6*+/− embryos supported a weaker coordination of LAO between epidermal layers (Figure S4A, see embryos #2,3 (LHS), embryo #4 and representative images, Figure 5b, *fz6+/−* Single SL). Conversely, *fz6−/−* suprabasal and periderm layers mostly coordinated their LAO with the bias of the basal layer, that is, towards circumferential/AV (Figure S4C), although, as mentioned, overall bias was less robust than wild‐type (Figure 5a and representative images, Figure 5b, *fz6−/−* single SL).

Disrupted coordination of LAO between epidermal layers was also apparent in *fz6+/−* skins which had thickened to two suprabasal layers. A slight overall bias in basal cell LAO towards circumferential/AV was not reflected in the adjacent suprabasal layer which favoured the AD quadrant, while periderm favoured circumferential/AV (*n* = 4 embryos, Figure 5c; Figure S5A). Representative images of *fz6+/−* epidermal layers are shown in Figure 5d (*fz6+/−* Two SL). Circumferential/AV bias, however, was more robust in *fz6−/−* basal cells in skins possessing two suprabasal layers compared to a single suprabasal layer, particularly in suprabasal and periderm layers, both overall (Figure 5c) and in individual embryos (Figure S5C: 6 of 7 mid‐flanks and representative images, Figure 5d, *fz6−/−* Two SL).

Altogether therefore, reduced Fz6 expression disrupts axial bias and coordination of epidermal LAO as the suprabasal layer is established and subsequently thickens. Coordination of LAO, however, although impacted by loss of Fz6 expression as the suprabasal layer is established, does not appear wholly dependent upon wild‐type levels of Fz6 signalling. In contrast, the switch in epidermal cell long axis orientation from circumferential/AV to longitudinal/AD is dependent on Fz6. Notably, *fz6−/−* embryos with a single suprabasal layer are strikingly similar to wild‐type embryos prior to establishment of a suprabasal layer: compare wild‐type Pre‐SL embryo #1 (Figure S1A) with *fz6−/−* single SL embryo #1 (Figure S4C). Finally, mirror symmetry of LAO across the ventral midline of *fz6* mutants appears less robust overall compared to wild‐type (Figures S4B,D and S5B,D), particularly in *fz6−/−* embryos with a single suprabasal layer (Figure S4D).

While robust trends in axial bias were observed in wild‐type and *fz6−/−* skins, variability in LAO was apparent between mouse embryos of the same genotype and between the left and right flanks of the same embryo. Embryo flanks from the same *fz6‐*mutant litters were scored based on their establishment of either a single suprabasal layer or two suprabasal layers. *Fz6*‐mutant flanks that did not exhibit a single suprabasal layer were excluded from this group; similarly, *fz6‐*mutant flanks which did not exhibit two suprabasal layers were excluded from this group. Scoring based on epidermal architecture might be expected to reduce variation. However, within the same litter, embryos are known to develop at slightly different rates (Miyake et al., 1996; Musy et al., 2018; Wanek et al., 1989) and one side of the embryo is also observed to develop faster than the other (Musy et al., 2018), which may account for the variation observed.

Finally, as wild‐type epidermal cell aspect ratio revealed E14.25 suprabasal cells (single layer) were more extended than at E14.5 (Figure 2a) and core‐PCP signalling is linked to epithelial cell shape (Hirano et al., 2022; Oozeer et al., 2017; Shi et al., 2014), epidermal cell aspect ratio between wild‐type and *fz6*‐mutant skins was also compared. No significant difference to wild‐type was observed; *fz6−/−* periderm cells were, however, significantly more extended than *fz6+/−* periderm cells (Figure S6).

### 2.5 An ‘outside‐in’‐oriented network of Fz6 protein expression connects basal, suprabasal and periderm layers

**FIGURE 6:**
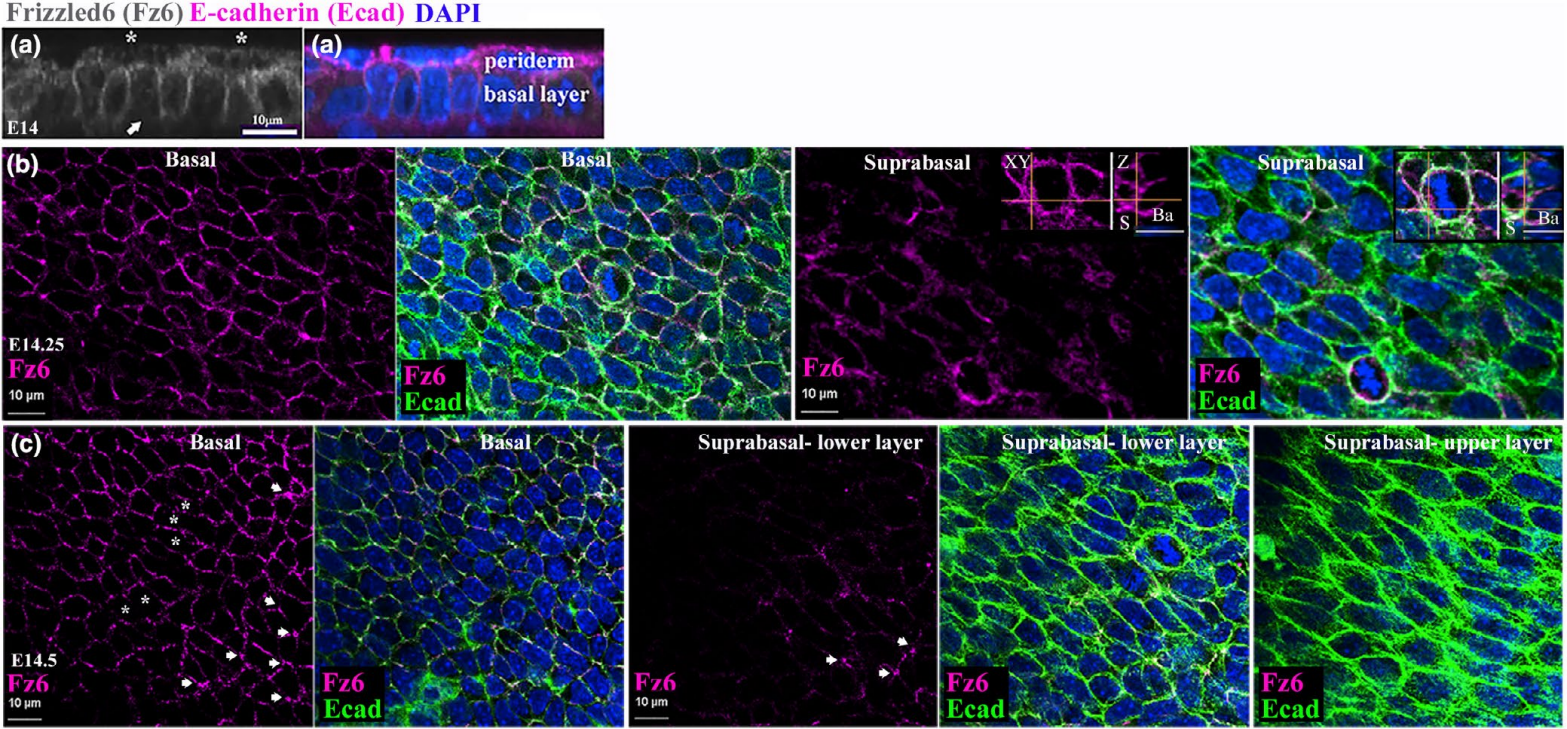
An ‘inside‐out’‐oriented network of Fz6 protein expression connects basal, suprabasal and periderm layers as the suprabasal layer is established. (a) Representative Z‐stack view, *n* = 3 images. Asterisks label periderm cells where Fz6 (greyscale) is enriched at lateral interfaces between periderm cells and between planar interfaces between the basal cell and periderm cell. White arrow labels absence of Fz6 along the basement membrane. E‐cadherin is shown in magenta. (b) XY views of wild‐type wholemount immunostained skins, representative image from *n* = 3 embryos, anterior is to the top, ventral is to the right. Ecad is E‐cadherin. Basal and suprabasal layers are shown. Fz6 enriches at the interface between the basal layer and suprabasal layer. Top right hand of suprabasal layer images show magnified XY view with associated Z‐stack views of mitotic suprabasal cell, Ba is basal layer, S is suprabasal layer. Top right quadrant reveals Fz6 expression within lateral interfaces of the basal cell which appears continuous with Fz6 expression within the dividing suprabasal cell. (c) XY views of wild‐type wholemount immunostained skins, representative image from *n* = 3 embryos, anterior is to the top, ventral is to the right. The area of epidermis shown is predicted to be switching LAO towards longitudinal. Basal and suprabasal layers are shown. White asterisks in image of basal layer label A‐P oriented asymmetric Fz6 expression across the basal layer, white arrows label Fz6 expression enriched within 4‐cell arrays or rosette type cell arrangements within the basal layer. White arrows in images of lower suprabasal layer label Fz6 enrichment at the centre of suprabasal layer rosette‐type cell arrangements sitting above basal layer rosettes.

**FIGURE 7:**
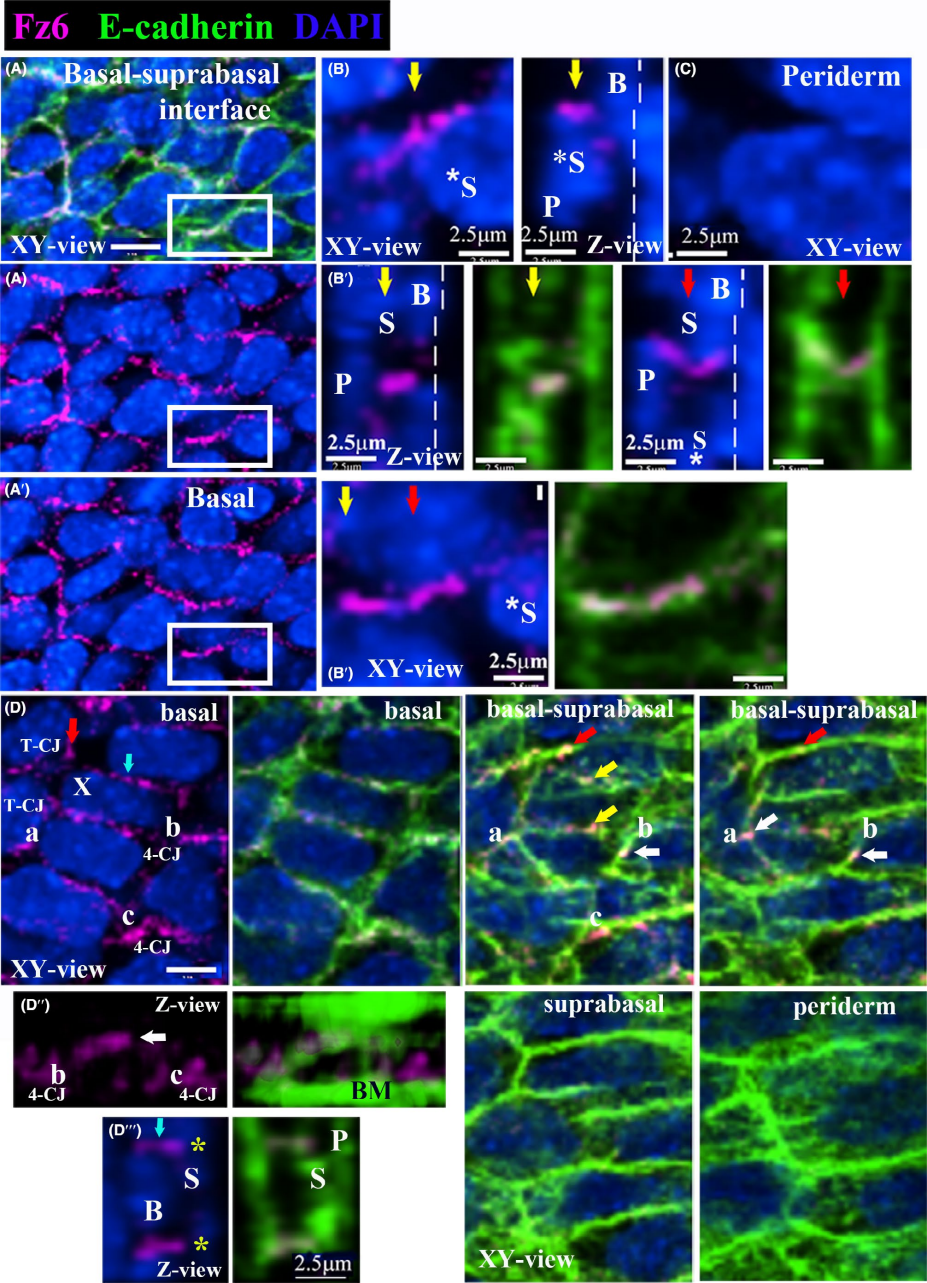
The ‘inside‐out’ network of Fz6 protein expression is associated with tri‐cell and four‐cell junctions. (A–D) XY and Z‐stack confocal images of wild‐type wholemount immunostained skins, representative images from *n* > 3 embryos. Unless otherwise indicated, scale bars are 10 μm. Anterior is to the top and ventral is to the right in all XY‐views. (A–C) XY and Z images showing (A) the basal‐suprabasal interface (A′) basal layer and (C) periderm layers. Magnified images of area highlighted by white box shown in (A, A′). (B, B′‐XY views) magnified areas from (A, A′). Coloured arrows represent position of Z‐views. *S labels suprabasal cell connected to basal cells via Fz6 expression. (B—Z views) Yellow arrows in (B) label position of Z‐view shown in XY panel (B). White dashed lines label basal lamina. B, basal S, suprabasal P, periderm. (B′—Z views) Yellow and red arrows in (B′) label position of Z‐view shown in XY panel (B′). White dashed lines label basal lamina. B, basal S, suprabasal P, periderm. *S labels suprabasal cell connected to basal cells via Fz6 expression. (D) Individual panels show XY‐views of basal layer, basal‐suprabasal interface, suprabasal and periderm layers. Blue arrow in basal layer panel labels position of Z‐view shown in (D″′), a, b, c label sites of Fz6 expression at intercellular junctions within the basal layer. T‐CJ is tri‐cellular junction, 4‐CJ is four‐cell junction. Coloured arrows in basal‐suprabasal interface panels label sites of interfacial Fz6 expression. (D″) 3‐D rendition (Volocity software) of D‐V oriented strand of Fz6 expression arising from four cell junction (4‐CJ) labelled (b) on image panel (D), (c) labels an adjacent 4‐CJ, white arrow highlights arc of Fz6 expression connecting to suprabasal layer. BM labels basal lamina. (D″′) Z‐view shown by blue arrow in (D). B, basal S, suprabasal cells, P, periderm. Yellow asterisks label intersection of Fz6 staining with suprabasal layer.

**FIGURE 8:**
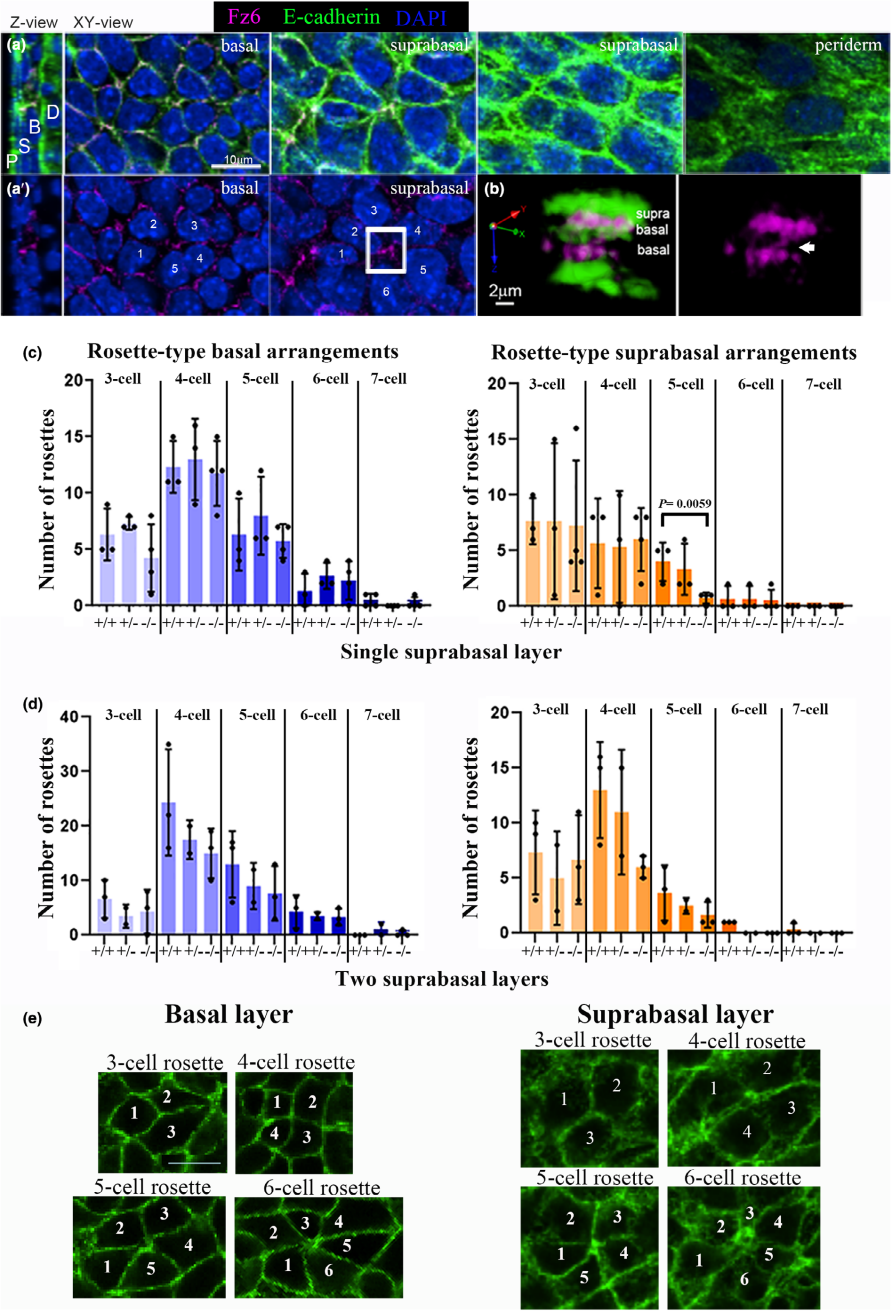
Rosette type cell arrangements are significantly reduced in *fz6* loss‐of‐function mutants. (a, a′) XY and associated Z‐views of wild‐type mouse embryonic epidermis which has thickened to two suprabasal layers. Individual panels show basal, suprabasal and periderm layers. Anterior is to the top and ventral is to the right in all XY panels. Z‐view, P, periderm S, suprabasal layers B, basal layer D, dermis. Numbers 1–5 label individual cells in 5‐cell rosette arrangements observed in both basal layer and overlying suprabasal layer. The suprabasal rosette sits directly above the basal rosette. White square box highlights Fz6 expression at the centre of the suprabasal rosette. (b) Volocity software generated 3D rendition of Fz6 expression within the basal and suprabasal rosettes shown in (a, a′). White arrow labels ‘inside‐out’ strand of Fz6 expression connecting the Fz6‐positive centre of the basal rosette (basal) with the Fz6‐positive centre of the suprabasal rosette. (c, d) Histograms show number of basal (blue) and suprabasal (orange) rosettes in mid‐flank epidermis from *fz6* knockout littermates. Mean number of rosettes is shown for each biological replicate where 4 images were available for analysis to ensure robust numbers of rosettes were scored for each embryo. +/+ denotes wild‐type, +/− denotes *fz6* heterozygote, −/− denotes *fz6* homozygote. Single suprabasal layer: +/+, *n* = 3 biological replicates from 3 litters, +/−, *n* = 3 biological replicates from 3 litters, −/− *n* = 4 biological replicates from 3 litters. Two suprabasal layers: +/+, *n* = 3 biological replicates from 3 litters, +/−, *n* = 2 biological replicates from 3 litters, −/−, *n* = 3 biological replicates from 3 litters. Statistical analysis was one‐way ANOVA, multiple comparisons, with Tukeys ad hoc test. (e) Representative examples of rosettes from basal (left) and overlying suprabasal (right) layers. Numbers 1–6 label individual cells in each rosette‐type arrangement, immunofluorescent stain labels E‐cadherin localisation. All images are to the same scale as scale bar shown for basal layer 3‐cell rosette (10 μm).

Coordination of LAO between epidermal layers was weaker overall in *fz6+/−* mutants. To gain insight into how wild‐type levels of Fz6 might coordinate epidermal long axis orientation in three dimensions, confocal images of wholemounted skins immunostained for Fz6 were examined in XY and Z planes. Surprisingly, Fz6 expression was found within the E14 periderm in addition to the basal monolayer (Figure 6a) and subsequently in the emerging suprabasal layer, including dividing suprabasal cells (Figure 6b and insets). Lower levels of Fz6 were also observed in the periderm at this stage (data not shown). As the suprabasal layer became established, Fz6 expression was increasingly refined in three dimensions: Fz6 A‐P‐oriented asymmetry emerged (labelled by white asterisks, Figure 6c basal layer) and Fz6 was found enriched to the centre of rosette‐type cell arrangements both within the basal and suprabasal layers (Figure 6c, white arrows). Notably, Fz6‐enriched suprabasal rosettes were sometimes found located directly below Fz6‐enriched basal rosettes (Figure 6c, white arrows). Further analysis revealed Fz6 planar asymmetry across A‐P oriented basal cell junctions coincident with ‘strands’ of Fz6 expression which connected to adjacent suprabasal cells (Figure 7A,A′—XY views; Figure 7B—*S, XY and Z views, yellow arrows; Figure S7B—white and yellow arrows). Indeed, from the basal lamina through to the underside of periderm cells, an ‘inside‐outside’ (radial) oriented pattern of Fz6 expression was observed (Figure 7B′, Z view, yellow and red arrows). Inter‐facial strands of Fz6 expression were also found associated with Fz6 expressing basal cell tri‐cellular junctions (T‐CJ) and four cell junctions (4‐CJ). Figure 7D (red arrows) highlights a T‐CJ adjacent to an interfacial enrichment of E‐cadherin which co‐localised with Fz6 puncta. Junction labelled (a) underlies a group of overlying cells in a rosette‐type arrangement with Fz6 enriched at its centre and elsewhere (white arrows nearby junction [a] and yellow arrows, Figure 7D). Junction labelled (b) is adjacent to Fz6 enrichment within an overlying D‐V‐oriented interface. A 3D rendition (Figure 7D″) highlights the position of the superficial Fz6 ‘strand’ compared to junction (b). Asymmetric A‐P oriented Fz6 expression (vertical blue arrow) within cell (X) is again shown here to directly connect to the underside of suprabasal cells (yellow asterisks, Z‐views, Figure 7D″′). Finally, in one instance a 5‐cell suprabasal rosette was found directly above a 5‐cell basal rosette (Figure 8a,a′). 3D rendition of Z‐stack images revealed ‘strands’ of Fz6 protein expression, perpendicular to the basal‐suprabasal interface, connecting the centre of the basal rosette with the centre of the suprabasal rosette (Figure 8b, white arrow right hand panel). Taken together these data support the hypothesis that Fz6 expression connects basal, suprabasal and periderm layers as the suprabasal layer is generated.

### 2.6 5‐cell suprabasal cell rosettes are significantly reduced in number in *fz6* homozygote knockout skins, which have established a single suprabasal layer

Rosette‐type cell rearrangements are employed across animal species to change cell orientation and alignment within the tissue plane (Blankenship et al., 2006). Given that immunohistochemical data above implicate Fz6 protein in a tissue organising principle which connects basal rosettes with overlying suprabasal rosettes, the hypothesis was raised that Fz6 may coordinate the circumferential to longitudinal switch in epidermal cell long axis orientation through a rosette‐based mechanism. As a first step towards addressing this hypothesis, the type and number of rosette‐type cell arrangements within basal and adjacent suprabasal layers from *fz6+/−* and *fz6−/−* skins were compared to wild‐type littermates (+/+). Basal layer 4‐cell arrays and 5‐cell rosettes (Figure 8e) were scored at similar levels in all littermates in nascent epidermis exhibiting a single suprabasal layer; however, a significant reduction in 5‐cell suprabasal rosettes in *fz6−/−* skins was found compared to wild‐type (Figure 8c). Although not significantly different, there was a trend towards fewer 4‐cell arrays and 5‐cell rosettes in *fz6* mutant basal and suprabasal layers once the suprabasal layer had thickened to two layers (Figure 8d). It was concluded that 5‐cell suprabasal layer rosettes are not efficiently generated when Fz6 is lost from the epidermis, supporting a role in the coordination of morphogenesis between basal and suprabasal epidermal layers.

## 3 DISCUSSION

Coordinated alignment of hair follicle orientation along the A‐P axis exemplifies how planar polarised asymmetry of core‐PCP proteins across opposing basal cell interfaces defines a structural axis of planar tissue polarity in mammals (Devenport & Fuchs, 2008; Wang et al., 2010). Recent evidence also demonstrates that core‐PCP proteins influence the ‘superficial‐deep’ (radial) architecture of the developing mouse epidermis (Panousopoulou et al., 2016; Hobbs & Formstone, 2023). Here evidence is presented for a novel, Fz6‐dependent tissue organising principle which plays a role in coordinating the long‐range alignment of epidermal cell long axis orientation (LAO) between epidermal layers, while the epidermal suprabasal layer is generated. Moreover, and unexpectedly, Fz6 is found to impact superficial epidermal layer morphogenesis just prior to establishment of the suprabasal layer.

Regarding the novel epidermal tissue organising principle, three key features can be proposed. Firstly, it was found that coordination of LAO between epidermal layers became more focussed in wild‐type as the suprabasal layer was established, implying that one role of the proposed tissue organising principle is to ensure the timely expansion of the developing suprabasal layer alongside the previously established basal and periderm layers. Indeed, the coincidence of circumferential/AV orientations for basal and suprabasal cells in wild‐type skins with a single suprabasal layer supports the notion that basal cells are subject to a tissue organising principle which determines basal cell LAO (Figure 1). Coordination of LAO between epidermal layers was disrupted however, when Fz6 levels were reduced (*fz6*+/−; Figure 5; Figures S4 and S5) but surprisingly, complete loss of Fz6 expression (*fz6*−/−) had less impact on mutant skin exhibiting two suprabasal layers than a partial loss (*fz6*+/−). A strong circumferential bias was found across all epidermal layers in the later stage *fz6*−/− skins (Figure 5). One plausible explanation is that other tissue‐level circumferentially oriented directional cues, which could be molecular, mechanical or adhesive in nature, can orient epidermal LAO in the absence of Fz6.

A second outstanding question relates to the molecular mechanism by which Fz6 orchestrates the robust coordination of LAO between epidermal layers. A central principle of core‐PCP signalling in fruit flies is that the asymmetric membrane expression of Frizzled/Flamingo/Vang receptors ensures robust, coordinated alignment of epithelial‐based structures along a specific tissue axis (reviewed by Strutt, 2009). Evidence presented here implies that planar polarised basal layer Fz6 asymmetry is coupled to a previously unreported, interconnected basal to periderm inter‐facial Fz6 expression, which, in turn, suggests the following compelling narrative: basal layer core‐PCP asymmetry defines the robust amplification of an underlying axis of tissue polarity which is then transmitted vertically (‘inside‐outside’) via the inter‐facial network of Fz6. This process most likely occurs in collaboration with other core‐PCP proteins. Indeed, LAO data for wild‐type embryos #2 & #3 (Figure S2A) could be interpreted as basal LAO shifting towards longitudinal in advance of overlying layers, which would support the narrative presented. We cannot however rule out the possibility that a tissue‐level polarity cue extends across the superficial periderm, which is then transmitted vertically from the periderm through to the deeper basal layer, that is, ‘outside‐in’. A combination of the two processes is also possible. Whatever the case, the source of Fz6 protein associated with suprabasal and periderm layers needs to be addressed in future studies.

A second key feature is the consecutive alignment of epidermal LAO along two orthogonal body axes, circumferential (anterior‐ventral) and longitudinal (anterior‐dorsal). Basal layer Fz6 planar asymmetry is observed to orient along both these axes at E14.25 (Figure 3c). A previous study also reported coincident circumferential and longitudinal orientation of PCP protein asymmetry across the E16 mouse epidermis (Oozeer et al., 2017). Moreover, French et al. (1976) and Bryant et al. (1981) proposed that amphibian limb epidermis exhibits a two‐dimensional map of positional values arranged along its longitudinal and circumferential axes. Devenport and Fuchs (2008) however, report a robust, long‐range A‐P oriented alignment of Fz6 asymmetry across the mouse epidermal basal layer at E14.5. Given that data from *fz6*−/− skins imply the presence of a circumferential tissue‐level cue at a scale large enough to orient LAO in the absence of Fz6, it is tempting to speculate that coherent, long‐range longitudinal (A‐P) oriented PCP is required at E14.5 to ensure LAO responds to longitudinal cues in a timely fashion. Indeed, competition between positional cues might explain the reduced overall robustness of LAO in wild‐type suprabasal and periderm layers at E14.5 compared to E14.25 (Figure 1). The developing anatomy of the epidermis supports a competing two‐dimensional map of positional values across the mouse epidermis: the epidermis is both enclosing over the spinal cord between E14.25 and E14.5 and closing in on the ventral midline (Figure 1a). Thus, tissue‐level circumferential cues could be generated by the process of SBW enclosure. Moreover, the wild‐type mouse embryo is lengthening longitudinally at an exponential rate around E14.5 (Panousopoulou et al., 2016).

Although not directly shown, the change in orientation of epidermal LAO described here between E14.25 and E14.5 could be deemed a rotation of at least 90° (circumferential to longitudinal). As longitudinal orientations were also observed in some embryos staged around E14, possibly because axial bias of LAO is not coordinated between opposing flanks, mid‐flank LAO could conceivably rotate through a complete 180° or rotation through 90° could reverse from longitudinal to circumferential. Importantly, rotation of LAO is delayed in *fz6*−/− mutants. Indeed, mid‐flank LAO patterns from *fz6*−/− mutants exhibiting a single suprabasal layer (E14.25) were strikingly similar to those of wild‐type embryos around E14. Conversely, basal cell patterns from *fz6*+/− littermates might be interpreted as a premature rotation of LAO towards longitudinal (observed in 3 of 5 *fz6*+/− embryos; Figure S4A). Strikingly, a similar phenotype was observed by Mirkovic and Mlodzik (2006) during investigation of *Drosophila* mutants of ommatidial rotation in the insect eye, which also requires core‐PCP protein signalling (Gaengel & Mlodzik, 2003). Thus, the hypothesis is raised that Fz6 levels impact the timing of the rotation of epidermal LAO from circumferential to longitudinal. What then is the cellular mechanism? The Fz6 enriched 4‐cell arrays and 5‐cell rosettes observed across the E14–E14.25 epidermis in both basal and suprabasal layers (Figure 3; Figure S3), alongside the significantly reduced number of suprabasal rosettes in E14.25 *fz6−/−* epidermis (Figure 8), paint a compelling picture of Fz6‐dependent cell arrangements within the epidermal plane driving the formation of cell arrays/rosettes in overlying layers, that is, along the orthogonal ‘inside‐out’ axis. However, the observed axial alignment of multiple, sequential Fz6‐positive cell arrays/cell arrangements across the epidermal basal layer (Figure 3) is equally striking, particularly because core‐PCP/Myosin II‐dependent mechanisms sequentially form and resolve rosettes during tissue rearrangements within the gastrulating *C. elegans* embryo (Xu et al., 2023).

A final feature of the tissue organising principle suggested by this study is the emergence of robust mirror symmetry of LAO at opposite mid‐flanks (right and left), once the suprabasal layer is established. Mirror symmetry of LAO is maintained during the axial switch from circumferential to longitudinal. E14.25 *fz6−/−* embryos (single‐SL) appear similar to E14 wild‐type (pre‐SL), that is, right and left mid‐flanks are not coordinated, which is consistent with a role for Fz6 in generating mirror symmetry of epidermal LAO, although the mechanism involved is unclear. The phenotype of *fz6+/−* mutants in this regard is less easy to interpret as it could be considered an indirect effect of the poor coordination of LAO observed between epidermal layers (Figures S4 and S5). Mirror symmetry of rotating epithelial tissues is not novel. In the E15.5 mouse epidermis, hair placodes exhibit a core‐PCP dependent pattern of local counter‐rotational cell flows occurring in an otherwise static epidermis (Cetera et al., 2018). Mirror symmetry is also a characteristic of the 90° rotation of ommatidia within the *Drosophila* eye (Wolff & Rubin, 1998), where both rotation and mirror symmetry depend on core‐PCP signalling (Weber et al., 2008). Finally, large scale counter‐rotation of lateral epiblast in the chick embryo occurs during primitive streak formation (Cui et al., 2005). Notably, apical contraction of planar polarised Myosin II cables leads to local cell rearrangements, which contribute to the counter‐rotational movements in the epiblast (Rozbicki et al., 2015).

An initial aim of this work was to understand how epidermal morphogenesis is directionally coupled to SBW closure. SBW closure defects were not however observed in *fz6* mutants which could be explained by the premature rotation of LAO in *fz6+/−* skins and the recovery of mirror symmetric circumferential LAO in *fz6−/−* skins. It is of considerable interest however that mirror symmetry across the ventral midline and coordination of LAO rotation from circumferential to longitudinal occurs as the suprabasal layer is generated. Moreover, ‘inside‐out’ Fz6 expression was first identified when the epidermis comprises a basal monolayer with overlying superficial periderm (Figure 6), and reduced Fz6 levels were found to impact superficial layer morphogenesis (Figure 3). Thus, understanding the mechanism(s) behind this previously unreported phenotype, whether it constitutes a defect in periderm morphogenesis and how it is related to the tissue organising principle proposed here, is essential. Important unanswered questions also remain regarding the role of peridermal E‐cadherin planar polarity. Going forwards, a careful analysis of the expression of PCP components across epidermal layers is required, together with investigation of LAO in multiple PCP mutants. Finally, establishment of live imaging techniques to monitor local cell rearrangements and cell shape changes, coupled to global changes in epidermal tissue shape, both within and between epidermal layers will be vital to fully understand the underlying molecular and cellular mechanisms involved.

Altogether this anatomical study raises exciting questions about the molecular and cellular mechanism(s) of three‐dimensional tissue polarity processes and how bi‐axial directional cues orchestrate epidermal morphogenesis, which may ultimately impact SBW closure in the mammalian embryo.

## 4 METHODS

### 4.1 Ethics Statement

Mouse litter sizes of 4–8 pups were expected with at least one homozygote mutant per litter thus it was determined that for each stage, no more than five pregnant females would be required to guarantee sufficient control and mutant embryos for experimental analysis. The same embryos were used for measurements of long axis orientation, aspect ratio and orientation of cell division across each epidermal tissue layer. Three fertile heterozygote males only for each genotype were maintained at any one time throughout the study. All mice were bred according to UK Home Office guidelines under Project Licence that was reviewed by the Animal Welfare and Ethical Review Body (AWERB) at King's College London before Home Office review, the Establishment Licence number for King's College London is X24D82DFF. Prior to individual studies taking place, a study plan was also reviewed within King's College London. Mice were held in individually ventilated cages at an average 21°C within a 12 h light/dark cycle with food and water provided ad‐lib. Mating trios (one male, two females) were set up and monitored morning and afternoon until females had plugged. At desired gestational age, pregnant females were killed via Schedule 1 (cervical dislocation) with all embryos killed by hypothermia and exsanguination.

### 4.2 Mice

The *fz6* mouse mutant has been described previously (Guo et al., 2004) and was bred on a C57BL6 background (RRID:MGI:2159769). All mice were between 8 weeks and 1 year of age. Genotyping of mice was performed using polymerase chain reaction (PCR). Timed mating was performed and 9 AM of the first day of plugging was taken as E0.5. Risk assessments were generated prior to experimentation.

### 4.3 Dissection of secondary body wall, wholemount immunohistochemistry and imaging

For each litter, embryos were assigned a date and a number and individually fixed in 4% formaldehyde in PBS for 1–2 h at room temperature and washed twice in PBS. Secondary body wall was dissected in one piece as an open‐book ventral peel. Briefly, the embryo trunk was dissected transversely just below the forelimb and just above the hindlimb. Parallel cuts were then made along the lateral sides of the spinal cord and the secondary body wall peeled away in one piece from the internal organs (anatomy of ventral peel is illustrated in Figure 1a). Ventral peels were then submitted to wholemount immunohistochemistry essentially as described by Devenport & Fuchs (2008).

Tissue was mounted in 70% glycerol and coverslips were sealed using nail varnish. Z‐stack images (0.25 μm steps) of mid‐flank epidermis from the right‐hand side and left‐hand side of each embryo were taken with a 60× (oil) objective using a Nikon A1R confocal microscope. Images were exported into Image J or Volocity (Perkin Elmer) for measurement and Volocity for 3D rendition. Immunohistochemistry images were manipulated in Adobe Photoshop CC 2019 using the *crop* function and annotated in Adobe Photoshop CC 2019.

The primary antibodies used were DECMA‐1 E‐cadherin (Uvomorulin; rat monoclonal, 1:4000, Sigma SAB4200684, RRID:AB_477600), E‐cadherin (mouse monoclonal, 1:1000, BD biosciences, 610182, RRID:AB_397581), Fz6 (mouse monoclonal, 10 μg/mL, R&D systems AF1526, RRID:AB_354842), pMRLC (pS19/pS20 rabbit polyclonal, 1:400, 600‐401‐416, Rockland, RRID:AB_217940) and Vinculin (mouse monoclonal, 7F9, 1:500, Invitrogen, RRID:AB_2532280); Celsr1 antibody (cyto‐N) was as described in Oozeer et al., 2017. Secondary antibodies (Life Technologies) were fluorescently labelled and used at 1:1000. DAPI was used at 1 μg/mL to stain nuclei.

### 4.4 Immunohistochemistry of paraffin wax and frozen sections

Keratin10 antibody (1:1000, Thermo‐scientific RKSE60), Keratin1 antibody (1:500; Covance AF109), Keratin 8 (rabbit monoclonal, 1:1000, ThermoFisher, MA5‐32118, RRID:AB_2809410) and Keratin 18 (mouse monoclonal, 1:100, ThermoFisher, MA‐06325) were used as primary antibodies on both wax sections (following antigen retrieval in 10 mM citrate buffer pH 6.2) and frozen sections using standard protocols. Controls were secondary antibody alone and for wax sections, no antigen retrieval. Secondary antibodies (Life Technologies) were fluorescently labelled and used at 1:1000. DAPI was used at 1 μg/mL to stain nuclei. Immunohistochemistry images were manipulated in Adobe Photoshop CC 2019 using the *crop* function and annotated in Adobe Photoshop CC 2019.

### 4.5 Analysis of epidermal cell long axis orientation, aspect ratio and orientation of cell division

*Measurements of long axis orientation, aspect ratio and cell division orientation* were performed manually. One mid‐flank image was analysed from each flank of each embryo (Figure 1a). Confocal images (Nikon A1R inverted) were taken from each mid‐flank. Right‐hand flank images (when viewed *en face*) were flipped horizontally when measurements were taken to ensure the same axial orientations were scored to the left‐hand flank. Epidermal cells surrounding and within hair follicles were avoided. Measurements of long axis orientation and aspect ratio were taken from epidermal cells in interphase. Each confocal image was divided into four quadrants. Measurements were taken from groups of cells in each quadrant. Measurements were then grouped based on whether the skin for an individual flank exhibited either (a) a basal monolayer and large rounded superficial cells (Pre‐SL; staged around E14), one complete suprabasal layer (Single SL; staged around E14.25) or two or more suprabasal layers (Two SL; staged around E14.5). Allocated embryo number was then translated into genotype and technical replicates for each flank of each embryo recorded individually within an excel sheet for each condition. Any *fz6* mutant embryos which exhibited a delay in suprabasal layer formation at E14.25 and E14.5 were not included for analysis.

*Long axis orientations* scored from each flank of each embryo were distributed into the appropriate bin widths defining axial orientation with respect to the ventral midline. A value of 1 was given to each measurement allocated to a specific bin. These values were summed for each bin and represented graphically as a percentage of the total number of measurements for each flank. Mean values for all embryos (biological replicates) for any one condition were generated and plotted as a percentage of the total number of measurements. Pre‐SL, basal cells and superficial cells were scored. Total cells scored for each group and for each condition are shown below.

Wild‐type Pre‐SL, *660 basal cells and 199 periderm cells*. Single SL, *777 basal cells, 506 suprabasal cells and 209 periderm cells*. Two SL, *501 basal cells, 249 suprabasal cells and 163 periderm cells*.

*fz6+/−* single SL, *1161 total basal cells, 564 total suprabasal cells and 538 total periderm cells*. Two SL, 715 *total basal cells, 418 total suprabasal cells and 155 total periderm cells*.

*fz6−/−* single SL, *511 total basal cells, 600 total suprabasal cells and 216 total periderm cells*.

Two SL, *511 total basal cells, 600 total suprabasal cells and 192 total periderm cells*.

A*spect ratio measurements* for each embryo flank were combined and the mean value calculated. Biological replicates were used for statistical analysis. Graphpad (Prism) was used for graphical representation of data. Each graph was pasted into Adobe Photoshop CC 2019 and annotated further. Statistical analysis was performed within GraphPad (Prism). Wild‐type: *E14; 131 basal cells, 90 periderm cells. E14.25: 100 basal cells, 100 suprabasal cells and 71 periderm cells. E14.5: 177 basal cells, 121 suprabasal cells and 60 periderm cells*.

*Measurement of orientation of cell division* including planar orientations was made in Volocity software (Perkin Elmer) for embryo number as described in Oozeer et al., 2017 or using Nikon software, NIS Elements version 6.10.02. For the latter, cell divisions were cropped and a 3D image generated. E‐cadherin staining along the basal membrane was used to identify the epidermal/dermal interface (basal lamina). Snapshots were taken from both (lateral view) sides of the dividing cell and the angle tool in Adobe Photoshop CC 2019 was used to measure the angle of cell division relative to the basal lamina. To ensure sufficient (n) for each condition, values for each embryo flank for each condition, for a single layer of suprabasal cells and two or more layers of suprabasal cells were combined. Planar orientations were binned as shown in Figure 2, each orientation was added into the appropriate bin as a value of 1. Graphpad (Prism) was used for graphical representation of data. Each graph was pasted into Adobe Photoshop CC 2019 and annotated further. Statistical analysis was performed within GraphPad (Prism).

### 4.6 Scoring of superficial cell number

Vertical and horizontal Z‐stack images were simultaneously generated using Nikon software from the top left, middle and bottom right regions of each confocal image in XY. For each orientation, the number of superficial cells along the length of the Z‐stack (250 μm) were counted. Mean values for each orientation and each region (six technical replicates) were calculated for each embryo (one biological replicate). Biological replicates (*n* = 5 for each condition) were graphically presented (GraphPad Prism). Each graph was pasted into Adobe Photoshop CC 2019 and annotated further. Statistical analysis was performed within GraphPad (Prism).

### 4.7 Scoring of rosette‐type cell arrangements

Four mid‐flank images taken from individual embryos were used to score rosette‐type cell arrangements to ensure sufficient (n) of rosettes for statistical analysis. Due to fewer confocal images available, biological replicates were only completed for (*n*) = 2 *fz6* heterozygote epidermis with two or more suprabasal layers (E14.5). Rosettes were scored manually and allocated into bins depending on the number of cells observed within the rosette‐type arrangement, that is, 3‐cell, 4‐cell up to 7‐cell. Graphpad (Prism) was used for graphical representation of data. Each graph was pasted into Adobe Photoshop CC 2019 and annotated further. Statistical analysis was performed within GraphPad (Prism).

## Supporting information


Data S1.

## Data Availability

The data that support the findings of this study are openly available in OSF digital at https://osf.io/yf7g8/overview. OSF: Public access to image files at https://osf.io/yf7g8/files.
