# Supplementary material for: A novel, PCP‐dependent tissue organising principle coordinating morphogenesis between embryonic skin epidermal layers
Source: J Anat. 2026 Jan 8;249(2):417–34. doi: 10.1111/joa.70099 (PMC13339934; doi:10.1111/joa.70099)
Supplement: Supplementary file 1 — Data S1. [file JOA-249-417-s001.pdf]

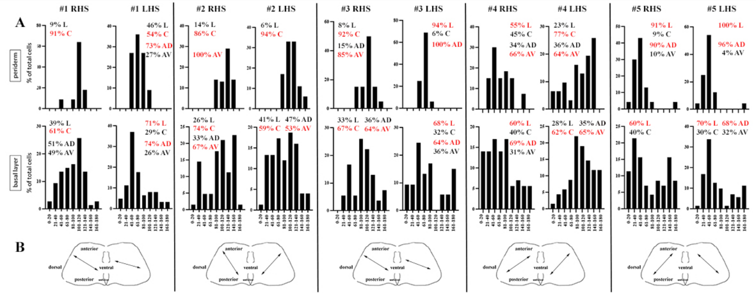

Fig.S1. **(A)** Plots of epidermal cell long axis orientation relative to the ventral midline taken from ventral body wall open book wholemounts immunostained with phalloidin and DAPI. Measurements taken from each right hand side midflank (RHS) and left hand side midflank (LHS) of individual E14 mouse embryos (n=5). Percentage (%) of longitudinal orientations (160 deg through to 70 deg) are shown (%L) above percentage circumferential orientations (70deg-160deg; %C). Percentage (%) of orientations in anterior-dorsal (AD 0-90deg) versus anterior-vental (AV 91-180deg) quadrants are also shown. Predominant axial bias is highlighted in red. **(B)** Schematics illustrate predominant axial bias for superficial (periderm) layer (black double headed arrow). Outer-most layer of superficial cells (periderm) were scored.

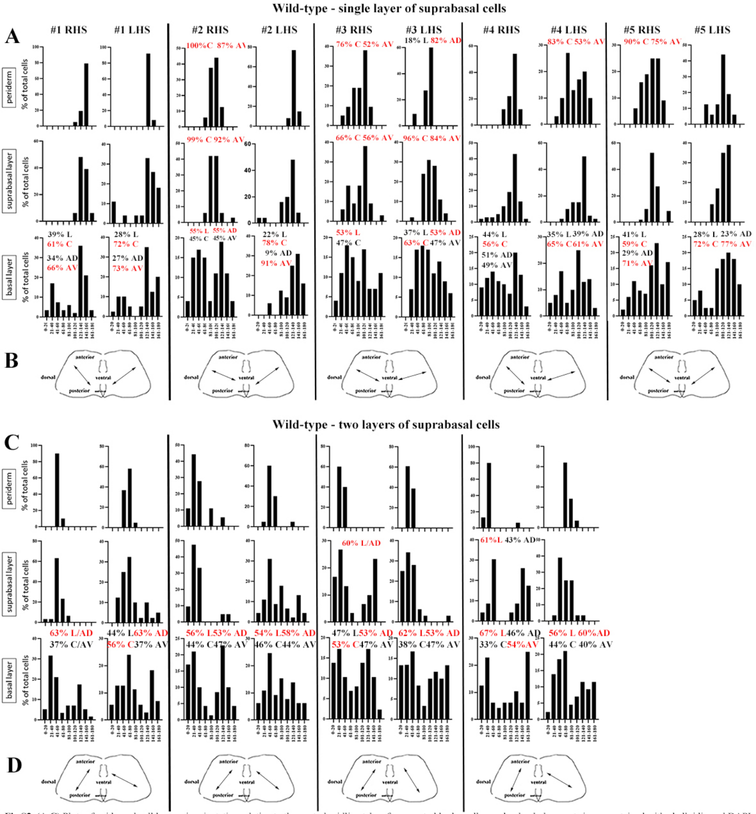

Fig.S2. (A,C) Plots of epidermal cell long axis orientation relative to the ventral midline taken from ventral body wall open book wholemounts immunostained with phalloidin and DAPI. Measurements taken from each right hand side midflank (RHS) and left hand side midflank (LHS) of individual wild-type mouse embryos **(A)** exhibiting a single suprabasal layer (n=5) or **(C)** exhibiting two suprabasal layers (n=4). Percentage (%) of longitudinal orientations (160 deg through to 70 deg) are shown (%L) above percentage circumferential orientations (70deg-160deg; %C) for basal cells. Percentage (%) of anterior-dorsal (AD 0-90deg) and anterior-ventral (AV 91-180deg) orientations are also shown. % values are shown for suprabasal and periderm layers when required to provide clarity. Predominant axial bias is highlighted in red. **(B,D)** Schematics illustrate approximate mean axial bias for suprabasal/periderm layers (black double headed arrows).

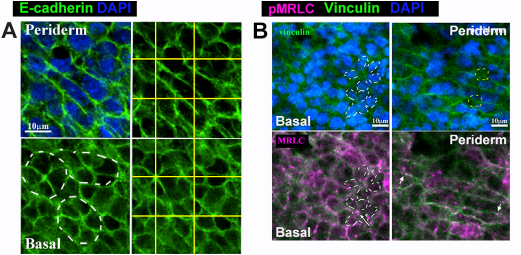

Fig.S3: Wholemount immunostaining of E14 epidermis, wild-type mouse embryos. Ventral is to the right in all images. Scale bars are shown. **(A)** Dashed white lines denote rosette-type cell arrangements in the basal layer. Right-hand side images are duplicates labelled with yellow grid lines to orient centre of basal rosettes with overlying planar polarised expression of E-cadherin. **(B)** Dashed lines outline rosette type cell arrangements in the basal layer and their localisation directly below planar polarised E-cadherin expression in the periderm.
Short white arrows label planar polarised pMRLC puncta, long white arrow in basal layer image labels pMRLC in the centre of a rosette, pMRLC is phosphorylated myosin regulatory light chain. Outer layer of superficial cells are shown and labelled as periderm.

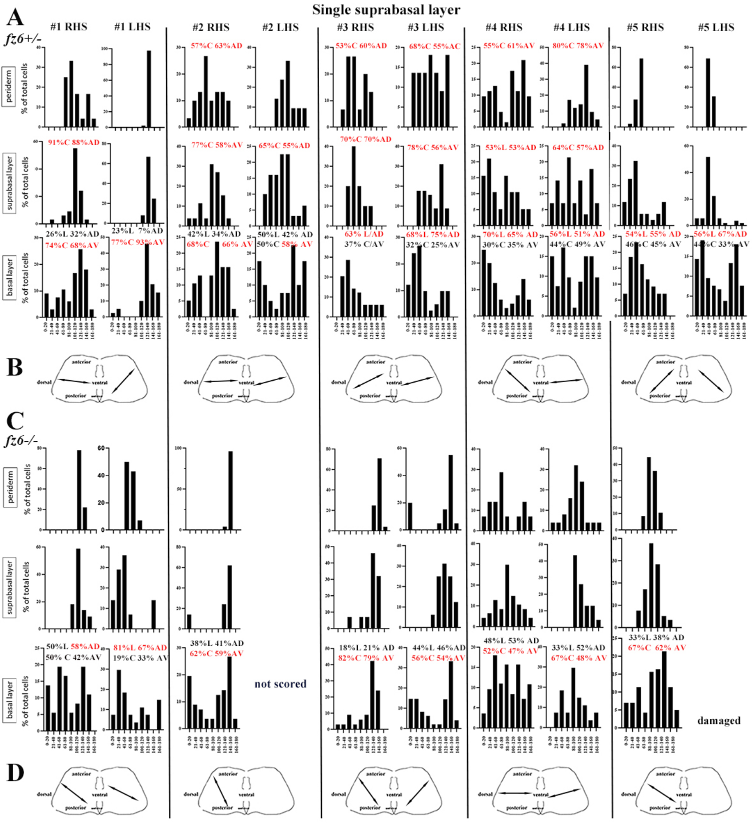

Fig.S4. Plots of epidermal cell long axis orientation relative to the ventral midline taken from ventral body wall open book wholemounts immunostained with phalloidin and DAPI. Measurements taken from each right hand side midflank (RHS) and left hand side midflank (LHS) of individual *fz6-/+* **(A)** and *fz6 -/-* **(C)** mouse embryos (n=5) for each condition. Percentage (%) of longitudinal orientations (160 deg through to 70 deg) are shown (%L) above percentage circumferential orientations (70deg-160deg; %C). % od AD and AV bias is also shown. Predominant axial bias is highlighted in red. % bias for suprabasal and periderm layers are shown only when they can provide clarity in terms of bias. **(B,D)** Schematics below histograms illustrate predominant axial bias for suprabasal/periderm layers (black double headed arrow). RHS of embryo 2 *fz6-/-* was not scored as the suprabasal layer was not esatblished. Overall, coordination of epidermal LAO is disturbed in *fz6+/-* mutants.

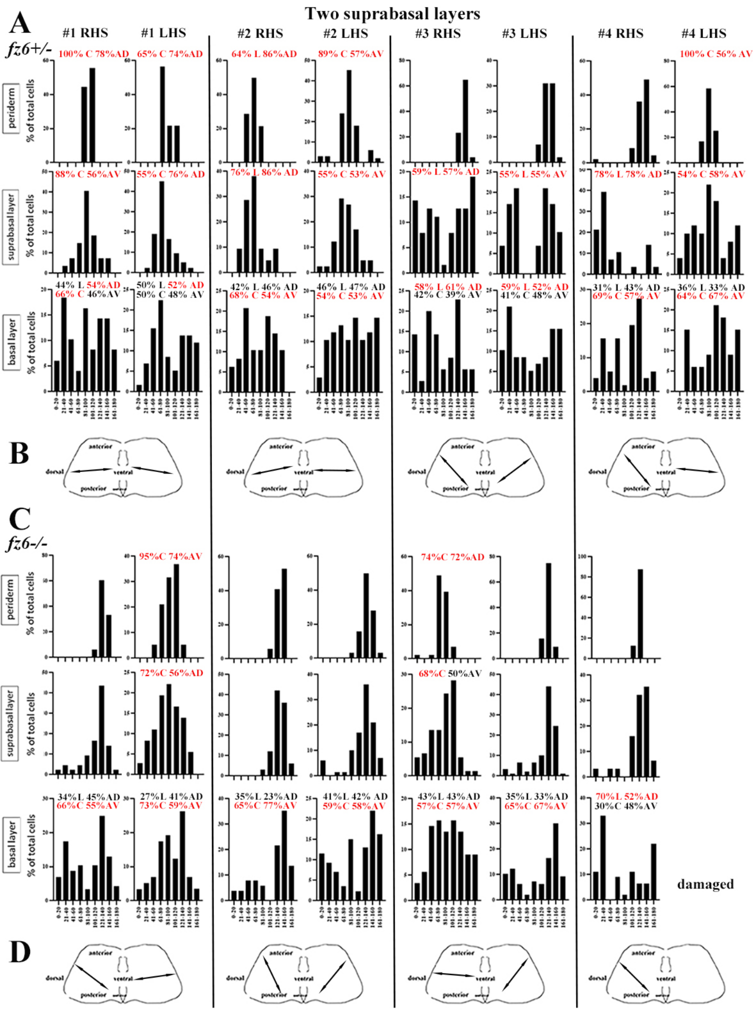

Fig.S5. Plots of epidermal cell long axis orientation relative to the ventral midline taken from ventral body wall open book wholemounts immunostained with phalloidin and DAPI. Measurements taken from each right hand side midflank (RHS) and left hand side midflank (LHS) of individual *fz6-/+* **(A)** and *fz6 -/-* **(C)** mouse embryos (n=4) for each condition. Percentage (%) of basal longitudinal orientations (160 deg through to 70 deg) are shown (%L) above percentage of basal circumferential orientations (70deg-160deg; %C). %AD vs AV also shown. For suprabasal and periderm layers, % bias is shown only where they provide clarity. Predominant axial bias is highlighted in red. **(B,D)** Schematics below histograms illustrate predominant axial bias for suprabasal/periderm layers (black double headed arrow).

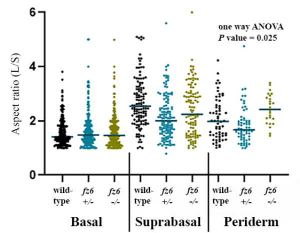

Supplementary Fig.6

Aspect ratio measurements for skins with a single suprabasal layer, n=5 biological replicates for WT, *fz6*+/- and *fz6*-/- skins. Statistical analysis was one way ANOVA with Tukeys ad hoc test. No significant differences were found for suprabasal cells although *fz6*-/- periderm cells were significantly longer than *fz6*+/- periderm cells.

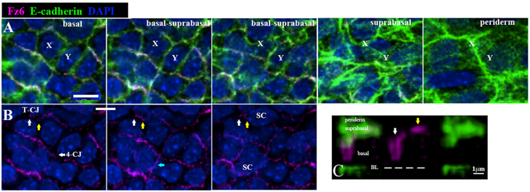

Fig.S7: **(A, B)** XY views of mouse epidermis, anterior is to the top and ventral is to the right. Panels show basal, suprabasal and periderm layers as well as basal-suprabasal interface. Letters X,Y denotes position of basal cell X and basal cell Y in relation to cells in overlying layers. **(B)** White and yellow arrows label XY position of Fz6 staining shown in Volocity software generated 3D rendition shown in **(C)**. T-CJ is tri-cellular junction, 4-CJ is four cell junction.

[illegible]
